# 

**Published:** 1893-04-29

**Authors:** 


					72 THE HOSPITAL. Apbjl 29, 18P3.
OUR NEW OFFICES. ?
428, Strand, W.O., will hencef:rth be the Office of thi-j Journal.
Notices of Births, Deaths, and Marriages, are inserted in
this column at a charge of 2s. 6d. for an announcement not exceeding
fonr lines.
NOTICE TO CORRESPONDENTS.
All MS., Letters, Books for Beview, and other matters intended foithe
Editor should be addressed Th? Editor, Tbe Lodge, Poechestib
Squabe,London,w.
The Editor oannot undertake to ruturn rejected M.S., even when
accompanied by stamped directed envelope.
Notice to Advertisers and Subscribers.
All Advertisements, Orders for Copies of Thb Hospital, and Businesa
Oommnninations should be addressed to The Publisher, not to the Editor,
Th* Hospital, <28, Strand, W.O.
The Cost of Subscription, post free, is as follows:?Per week, 2id.>
per quarter, 2s. 6d. ; per half-year, 5s. ; per annum, 8s. 6d.
Foreign ;~Per annum, 10s. 83.; cay able, in all cases, in advance.
" Burdett's Hospital Annual," 1893.
Fourth year of publication. 700 pp. Boards, gilt lettering. A most
complete sruide to British, Colonial, Indian, and American Hospitals,
Dispensaries, Nursing &nd Convalescent Institutions, and Asylums.
Prioe5s. Published by The Scientific Press (Limited), 423, Strand,
W.O.
NOTICE.?The Index for Vol. XII. fending1 September
1893) is on sale at the Office of this Journal, price
2Jd., post free.
Scraps and Gleanings.
Oveh 12 0Q0|person? are found employment for in the theatres of ctsr
Metropolis.
De. Reidal, physician to the King of Spain, visita hiB Royal patiemt
three*times dnrirg the day.
There are 950 miles of telephone line between New York and Chioigo;.
this is the greateit length ex> ant.
The authorities of W?ltham Abbey have made arrangement} to erect
an Infectious Diseases Hospital.
The first prise for the beat examination to the University cf Chicago
was pasted last Christmas by a young coloured lady.
The site for the proposed infectious diseases hospital at Northaa
continues t J cause much discussion among the ratepayers.
ARussiah peasanthas just died aged 120 years. One of his sons had
attaine i the age of 92, ana his father was ISO when he died.
The electric lighting of the Chicago Exhibition will have to be pro-
vided with 15,000,000 feet of insulated wire in order to distribute the
current.
It is satisfactory to be able to ktate in the interests of the Swansea
Hospital that the financial proceeds of the annual ball recently held
amount to ?93.
The Swedes h va a curious method of treating drunkards} these
offenders are imprisored forthwith, and during their detention are fed
on bread and w ite.
The annual meeting of German Naturalists and Medioal man will bo
he din Sertamb r at Nuremberg. Tie addresses will ba delivered by
Professor V. Helmholz.
The report of the anual meeting of the Horsham Cottage Hospital was
a most favourable one. The Committee's report dealt with a period ot
aiximonths, during whioh no death had occurred. There was a.
balance in hand ot ?119 13].
_PnoT?ST3 against the proposed site, at Canterbury, of an Infectious
Diteases Hotp.tal, continue to increase in nnmbers. The site is objected
to as interfering with, and virtually destroying, one of the mos4
frequented walks round Canterbury.
We ara glad to be able to report that the Duke of "Westminster haj
given ?U0 towards the co>t of sending to every clergyman in the
kirgdom a copy of the first number of " Temporal Welfare," the crgan
of the Church of Ecglaid Sanitary Association,
Votes are urgently needed for Qeoige Edward Ferguson, who is a
candidate for election. May, 1893 (last chance), at the Royal Meiioal
Benevolent College, Epsom. Proxies will be thankfully received b/
Mrs. K. Ferguson, Olendale, Ta&well Road, Scuthsea.
The Coventry Provident DiBfomary has now 25,000 members. Thia
establishment was bmlt for the avowed purpose of enocuraging thrift
among the poor; but it appears that many of the members belong to a
class who have no right to derive benefits from a provident institution.
The bazaar held recent'y in the Eye Infirmary at Sunderland proved
a great financial snccess, as well as enabling visitors to insocet tne new
buildings, ?200 was added to the lunds of the institution * through
receipts from the two dayb' sale, and the existing mortgage was
paid off.
The Editor of a Budapest newspaper hai given 250,000 florins on the*
death of hia Wife, for the foundation of a children's tojpital for that
city. Thia generous gift comes from one of tte Jewish faith, indeed,
few but Jewa ever devote sush sums to purpose* of that charitable kind
in Hungary.
Por the Book World of Medicine and Science, and Medical Vacancies and Appointments, see pases VIZI., IX .
and X., overleaf.
FIRST IMPRESSIONS OF BOOKS RECEIVED.
Lit is requested that all books intended to be notioed in this column
may be sent to the Editor, at the Office, 428, Strand, W.O., not later
than Tuesday evening in each week.]
The Hunterian Oration. By Thomas Bryant, F.R.C.S.E.,
&c., President of the Boyal College of SnrgeonB of
England, Consulting Surgeon to Guy's Hospital.
(London : Printed by Adlard and Son.)
One interest attaching to this oration is the fact that it
was delivered on the centenary of Hunter's death, and in
the presence of T.R.H. the Prince of Wales and the Duke
of York. Mr. Bryant deals with Hunter as a thinker and
worker, as a biologist, and as a surgeon, and contrives to
impart a fresh interest to his treatment of a well-worn
theme.
Clinical Lectures on Abdominal Hernia. By William
H. Bennett, F.R.C.S., Surgeon to St. George's Hospital.
(London, Longmans, Green, and Co., 1893.)
Hernia has always been * favourite theme for clinical
lectures. The importance of the subject is great, and it is
only equalled by the variations that are met with in cases at
first sight alike. Everything continues to make Hernia a
ohosen theme, and Mr. Bennett has followed in the foot-
steps of many another lecturer on clinical surgery in
making it his subject at several meetings of his class.
The leotures deal rather with the unusual phases of the sub-
ject than with the commonplace and familiar. Many
interesting and curious cases are recorded, and Mr. Bennett
discusses most of the important points that arise in the
THE HOSPITAL, Apeil 29, 1893.
Medical Vacancies and Appointments.
APPOINTMBW3TS.
W. H. Ackland, M D.St. And., M.R.C.S.Eng., reappointed
Medical Officer of Health for the Bideford Urban Sanitary
District. J. C. Bates, M.R.C.S., L.R.C.P.Lond., appointed
Medical Officer for the No. 1a, Upper Norwood District of the
Croydon Union. Q. H. A. C. Berkeley. M.B., B.C.Cantab,
M.R.C.S.Eng., L.R.C.P.Lond., appointed House Surgeon to the
Middlesex Hospital. A. A. Blackiston, M.R.C.S.Eng., re-
appointed Medical Officer of Health for the Glastonbury Urban
Sanitary District. C. H. Bond, M.B., Ch.M.Edin., appointed
Pathologist and Fourth Assistant Medical Officer to the London
County Asylum at Banstead. W. E. Bond, M.R.C.S., L.R.C.P.,
appointed Assistant Medical Officer to the Stockport Infirmary.
J. R. Bradford, M.D., M.R.C.P.Lond,, appointed Assistant
Physician to the National Hospital for the Paralysed and
Epileptic, Queen Square. N. B. Clowes, M.R.C.S., L.R.U.P.,
appointed Medical Officer to the Reading Dispensary. W. S.
Colman, M.D., M.R.C.P.Lond., appointed Registrar and
Pathologist to the National Hospital for the Paralysed and
Epileptic, Queen's Square. H. M. Cullinan, L.R.C.S., L.R.C.P.I.,
appointed third Assistant Medical Officer and Pathologist to the
Richmond District Lunatic Asylum. J. Currie, M.B., C.M.
Glasg., appointed Acting Medical Officer to the Madras Railway
Company, Madras. R. Erskine, M.D.Q.U.I., reappointed Medical
Officer of Health to the Camborne Local Board. J. A. Fisher,
M.R C.S., L.R.C.P.Lond., reappointed Medical Officer of Health
for the Garston Urban Sanitary District. F.J. Fletcher, L.S. A.,
reappointed Medical Officer for the Burton Coggles District of
the Grantham Union. J. Freeman, M.R.C.S., L.R.C.P.Lond.,
appointed Anaesthetist to the Bristol General Hospital.
April 29, 1893 THE HOSPITAL. xi
MEDICAL VACANOIES AND APPOINTMENTS?(continued).
VACANCIES.
Vh? following ? ?? ncies are annonncod this week, the amount of
salary in eaoh olse t>?>ng friven after the name of the institution:
Resident Medical Officer"
HirminghEm General Dispensary, Birmingham. Resident Surgeon fee
the Hiphrate Branch: do*bly qualified. Salary ?150 per aonum,
with allowanci for o?b hire, an* furniihed rooang, fire, lights, ana
attendance. Applications to Alex. Forrest, Secretaty, by May
Dcrby'oounty Asylum. Resident 0 iniral Aspittint. Appointment for
six months. Beard, attendance. &o., provided. Applications to
the Medical Superintendent, County Asylnm, MicV] rover, De^by.
General Hospital, Birmingham. Assistant Ofcstetrio Officer. Resident
Surgical Offiocr. Salary ?180 per annum, with reudanoe, board,
Kitnaid waBhing. Applications to the Hons* Governor by Aoril 29th.
General Infirmary, at Leeds, Thr?e House Surgeons. Two fo? a period
of twelve months aid one for six months. Bosrd. lodging, and
washing provided. Applications ta Mr. Littlew od, Seoretary to
the Faculty, General Infirmary, Leeds, by April 27tb.
Reyal Berks Hospital, Reading. House Surgeon. Salary ?80 per
annum, with board and lodging. Applications to the Secretary by
May let,
Hon.-Resident Medical Officers.
Board of Works for the Lewishsm Di?trict. Hospital Medical Officer
for the Infections Diseases Hospital at Hitber Grwn. Applica-
tion". endorsed " Hospital Medics.1 Officer," to Edw Wright, Olerk
to the Bjard, Board of Works Office, Catfard, by May 2nd.
Dnnsbaughlin Union, Dunboyne Dispensary. Medical Officer. Salary
?110 i?r atnnm, wth ?15 as Medical Offir er of He&Hh, exclusive of
registration atd vaccination fees. ApplicatioLs to Mr. Lawi ence
Ward, Hororary Secretary, Tfce Grove, Clonte, co. Meath. Election
on April 28th,
London Hospital. Whiteehapel Road, E. Medical Registrar. Salary
?100 per annual. Application* to the Home Govimor by Mny 6th.
Nations 1 Hospital for Diseases of the Heart a^d Paralysis, Soho Square,
W. Two Olinicjil Assistants; donbly qualified. Application to the
Secretary by April 29th.

				

